# Thermal Conductivity Gas Sensors for High-Temperature Applications

**DOI:** 10.3390/mi15010138

**Published:** 2024-01-16

**Authors:** Nikolay Samotaev, Boris Podlepetsky, Mikhail Mashinin, Igor Ivanov, Ivan Obraztsov, Konstantin Oblov, Pavel Dzhumaev

**Affiliations:** Institute of Nanoengineering in Electronics, Spintronics and Photonics, National Research Nuclear University MEPhI (Moscow Engineering Physics Institute), Kashirskoe Highway 31, 115409 Moscow, Russiapsdzhumaev@mephi.ru (P.D.)

**Keywords:** thermal conductivity, ceramic, platinum wire, micromachining, laser micromilling

## Abstract

This paper describes a fast and flexible microfabrication method for thermal conductivity gas sensors useful in high-temperature applications. The key parts of the sensor, the microheater and the package, were fabricated from glass-coated platinum wire and the combination of laser micromilling (ablation) of already-sintered monolithic ceramic materials and thick-film screen-printing technologies. The final thermal conductivity gas sensor was fabricated in the form of a complete MEMS device in a metal ceramic package, which could be used as a compact miniaturized surface-mounted device for soldering to standard PCB. Functional test results of the manufactured sensor are presented, demonstrating their full suitability for gas sensing applications and indicating that the obtained parameters are at a level comparable to those of standard industrially produced sensors. The results of the design and optimization principles of applied methods are discussed with regard to possible wider applications in thermal gas sensor prototyping in the future. The advantage of the developed sensors is their ability to operate in air environments under high temperatures of 900 °C and above. The sensor element material and package metallization were insensitive to oxidation compared with classical sensor-solution-based metal–glass packages and silicone MEMS membranes, which exhibit mechanical stress at temperatures above 700 °C.

## 1. Introduction

Gas sensors using environmental changes in thermal conductivity have historically been used in industrial applications for the measurements of high concentrations of flammable and inert gases. Such applications include controlling the level of hydrogen in electrolysis or detecting high concentrations (above potentially explosive levels) of methane in mining operations. Nowadays, with the development of the hydrogen energy field, the need for thermal gas sensors is increasing in automotive applications. The working principle of thermal gas sensors is similar to catalytic sensors [1], except there is no chemical reaction with oxygen. Thermal conductivity sensors comprise two microheaters, one of which is exposed to the target gas (the detector), the other of which is sealed inside a chamber containing air (the compensator). Both microheaters are heated and run in the same type of circuit as catalytic sensors; thus, they exhibit similar electrical characteristics and can be used in the same Wheatstone–Bridge circuits. When the detector microheater is exposed to a gas whose thermal conductivity is significantly different from that of air, the rate of heat loss from the microheater will change, as will its resistance. This measured change is compared with the compensator microheater values. Thermal conductivity sensors are most often used to detect gases with low molecular weight, which have greater thermal conductivities than that of air, consequently giving the highest response. This also means that some gases, such as oxygen, nitrogen, and carbon monoxide, cannot be measured, as their thermal conductivities are too similar to that of air. These differences can vary with the gas temperature. The main applications for methane-calibrated thermal conductivity sensors are coalmines and natural gas monitoring. Concentrations of methane well over 50% by volume can be found in fissures in coal seams. The other main application for thermal conductivity sensors is in the monitoring of “light” gases such as helium and hydrogen. Helium can be found in various industrial processes; hydrogen can be present in fuel cells and battery charging applications. A more detailed overview of the theory and practice of using thermal gas sensors is presented elsewhere [1].

If the physical principle of operation is the same for all thermal conductivity sensors, then their distinctive features are the design and manufacturing technology. Thermal conductivity sensors are available with a wide variety of working voltages and power levels. In general, higher-power devices are used in fixed systems, and lower-power versions are utilized in portable equipment.

Classic “Pellistor” types of sensors, wound from platinum wire 20 µ or thicker, are the most powerful sensors [2]. Higher-power sensors, which typically use standard stainless steel packages 20 mm in diameter [3], have porous filters on top for explosive protection. The main target of the package is to seal the compensating element fully. A loss in signal with exposure time will be observed if the target gas is allowed to reach the compensating element. Changes in the humidity and pressure of the target gas will affect the rate of heat loss from bead-type microheater detectors; hence, the sensor will read zero. In practice, however, the effect is not significant. To achieve isolation, the bottom of the package should consist of bushing epoxy resin, which enables operation at ambient temperatures between −40 °C and 55 °C; such packages should not be used outside this temperature range (meeting the established certification demands [3]).

The second type of technology is low-power sensor chips, which consist of a silicon frame with a silicon nitride membrane. In the center is a microheater with a thermopile measuring its temperature. The chip measures the thermal conductance between the ambient atmosphere and the center of the membrane. A silicon cover on top of the membrane and a welded-on package cap with a filter suppresses flow sensitivity [4]. Typical for low-power sensors is the use of metal–glass packages; TO-8 or TO-5 are widely used in microelectronics [4,5]. Here, the convenience of installing the chip into the package using ultrasonic welding and gold microwire is the primary concern. For such types of packages, the construction operating temperature is wider: −250 °C to 150 °C [5]. This can explain the absence of organic compounds (epoxy resin) that are used in higher-power devices, although real producers of these packages advise a slightly different operating temperature range: −65 °C to 155 °C [6]. Extensive information about the package properties is scant; therefore, in this study, we verified the operating temperature limits stated here for low-power sensors by performing tests in a kiln.

**Figure 1:**
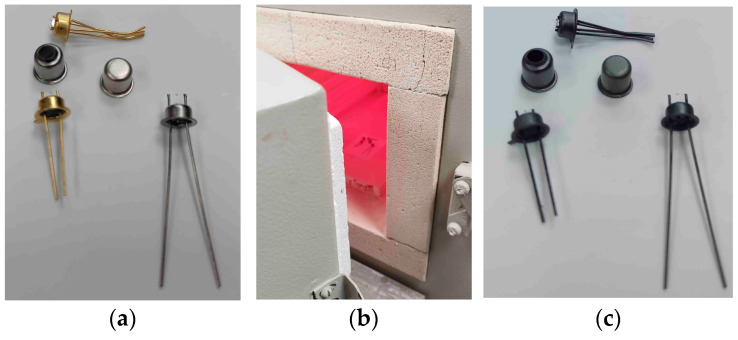
High-temperature gas sensors in TO-18 packages: (**a**) Photo of sensors package before firing (cap of packages disassembled); (**b**) Photo of open kiln door during 10 min firing packages @ 850 °C in air; (**c**) Photo of packages view after being in the kiln at high temperature; it is possible to see total oxidation of package plating compared with before firing.

As an experiment, we selected packages used for serial micropower thermal conductivity sensors. The initial photo of the packages before they were heated in the oven for 10 min at 850 °C under an air atmosphere is shown in Figure 1a. The TO-18-type packages [6] with different coatings (electroless nickel, 3 mkm thick + 0.1 mkm thick immersion gold, called the ENIG coating; electroless nickel, 3 mkm thick + 3 mkm thick, called electroplating gold; and electroless nickel, 3 mkm thick) and caps made by Kovar (nickel–cobalt ferrous alloy) and pure nickel were taken as samples for testing. The test results are presented in Figure 1c—there were predictable increases in the total losses of coatings and electrical contact with pins. Additionally, it became clear that at the operational temperature, the silicon membrane with a microheater (mounted on one of the packages; the membrane was taken from a previous study [7]) collapsed from thermal stress, whereas the platinum microwire heater retained its functions, although in some cases became disconnected from the package pin. The described experiment indicated that standard thermal conductivity sensor solutions are not suitable for high-temperature processes involving environmental ambient air. In a humid environment, the Kovar alloy is susceptible to corrosion and requires protective anti-corrosion coatings. Typically, for this purpose, the contact pads of such electronic packages are made of nickel-plated alloy. However, the coating in the form is not a panacea because such coatings are designed for short-term temperature increases up to 400 °C in order to use the thermal compression of the gold wire. In addition, the most popular type of ENIG coating contains phosphorus, which at 250 °C begins the process of recrystallization [8] and the initialization of the destruction of the functional coating of the package. To overcome this gap, it is necessary to use other materials that are resistant to oxidation in air. In electronics, these are alumina ceramics and thick-film metallization based on precious metals—gold, platinum, and silver. As evidence of stability, the listed materials are widely used in the LTCC process with technology temperatures of 850 °C in air ovens vs. HTCC materials using hydrogen ovens [9] for the protection of metallization material against high-temperature oxidation during firing. Note that ENIG finish coating can be used in HTCC packages for high-temperature gas sensors.

The motivation of this paper is to present novel developments in thermal conductivity gas sensors for high-temperature applications. The main aim of this research is to demonstrate a rapid prototyping method for thermal gas sensors in a miniature ceramic SMD package and the reproducibility and stability of manufactured sensor properties based on using production materials (alumina, platinum, silver, gold, and glass) in high-temperature technology processes (up to 1000 °C). Laser micromilling of fired ceramics gives us fine results in previous work with catalytic-type gas sensors [7]. Field-effect gas sensors [10] and metal oxide gas sensors [11] are used at high operation temperatures and are capable of working in harsh environmental conditions.

## 2. Materials and Methods

**Figure 2:**
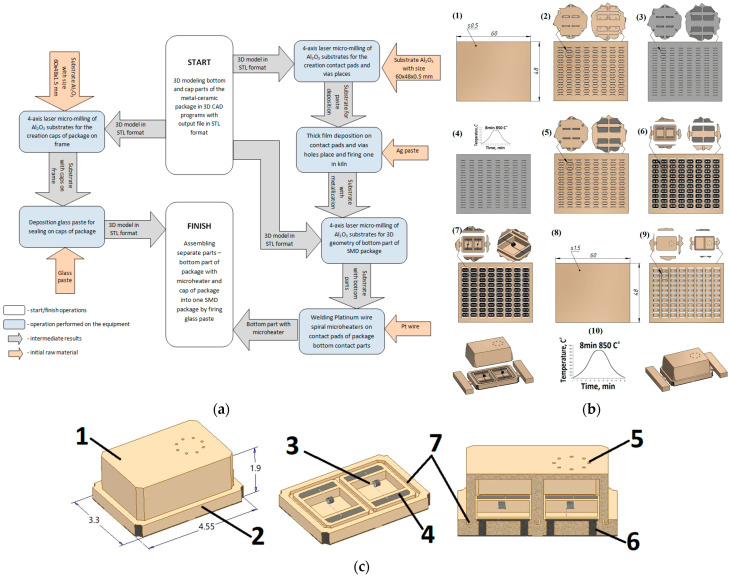
The technologies used for rapid prototyping thermal conductivity gas sensors (dimensions are given in mm): (**a**) Full flowchart for rapid prototyping of the sensors; (**b**) The technology operations with ceramic substrates in the process of its fabrication. The start of flow chart is from left to right and direction of technology operation is from top to down. (1) Initial ceramic substrate for bottom of package, (2) substrate with holes for contact pads and vias after laser micromilling, (3) Ag paste screen-printing on substrate, (4) deposited Ag paste firing on substrate, (5) polishing Ag metallization after on substrate, (6) substrate after laser micromilling 3D geometrybottom part of package, (7) welding sensor element on bottom part of package, (8) initial ceramic substrate for cap of package, (9) substrate after laser micromilling 3D geometry cap of package, (10) final assembling of sensor by gluing on glass both parts of package; (**c**) 3D model of gas sensor in the SMD package using for fabrication: 1—Al_2_O_3_ ceramic cap of package; 2—Al_2_O_3_ ceramic bottom of package; 3—Pt-wire micro heater; 4—Ag contact pads; 5—holes in package cap for gas diffusion on active sensing element; 6—vias hole between inside and outside contact pads filling with Ag metallization; 7—tongue-and-groove joint for filling with glass.

To fabricate the gas sensor, we used the technologies presented in Figure 2a,b. We developed a 3D model of the sensing device by using 3D modeling software Autodesk Inventor CAD. The sensor package size is 3.3 × 4.5 × 1.9 mm^3^. To reduce the sensor prototype cost, 96% Al_2_O_3_ monolithic ceramic was used to manufacture the package of the sensor. The special 20 W fiber laser with tunable pulse duration in the range of 50–200 ns and a wavelength of 1.064 μm, controlled by specially produced software [11], was used to fabricate different parts of the developed sensor. This approach allowed us to combine the process of micromilling with a comparison of the fabricated devices, its geometrical parameters within the 3D model, and the achieved quality after its fabrication.

### 2.1. Platinum Microheater

**Figure 3:**
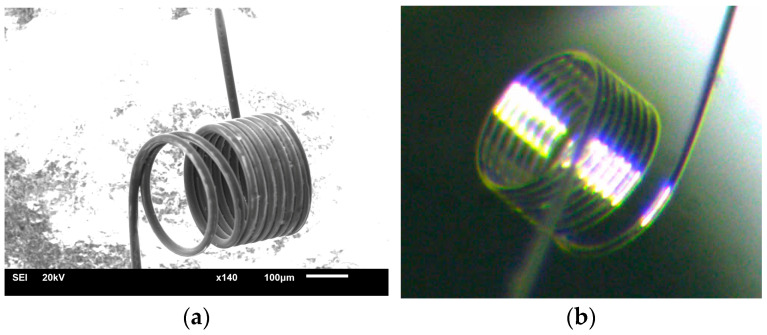
Platinum microheater: (**a**) The microheater in the form of a platinum spiral obtained by JEOL JSM-6610LV Scanning Electron Microscope (JEOL, Ltd., Akishima City, Japan); (**b**) Optical image of a microheater in the form of a platinum spiral.

The platinum heater (Figure 3) is a spiral with a diameter of the internal metal core of 10 μm with a 2 μm glass-coating and 9–10 turns with a diameter of 200 μm. This type of heater was chosen because of its high stability as a microheater for catalytic sensors [12,13,14,15]. In the glass-coating method in process of platinum wire fabrication, known as “Taylor-Ulitovski process” [16], the platinum is placed into a glass tube (typically of high-silica glass) closed at one end and afterward heated by a high-frequency inductor. The tube is heated until metal part is molten liquid and the glass of the tube has softened enough to be drawn into a fine glass capillary with a metal core. Thus, a microwire is formed, consisting of a central metallic core and a continuous glass shell. High-silica glass insulation allows isolation of the platinum wire from the environment, thereby stabilizing its thermal and electrical parameters. The glass insulation not only improves thermal stability of the microwire but also helps prevent the interaction of platinum with ambient atmosphere. An optical and SEM image of a microheater in the form of a platinum spiral used in the work is presented in Figure 3.

### 2.2. Laser Fabrication of Ceramic Package for Sensor

**Figure 4:**
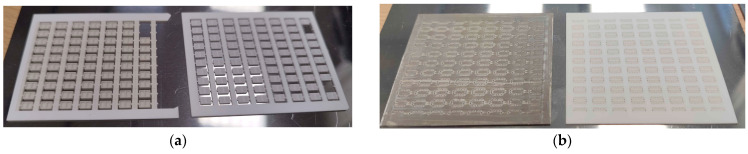
Ceramic Al_2_O_3_ substrates after laser micromilling: (**a**) Photo with front and back view of substrates with package cap; (**b**) Photo with front and back view of substrates, which have contact pads and via holes deposition of Ag thick film. The left substrate shows deposited and dried Ag paste, and the right image indicates the same substrate immediately after laser micromilling before Ag thick film via screen-printing.

The prototyping SMD packages were realized by laser micromilling technology with Al_2_O_3_ ceramic substrates (96% alumina) in the size of 48 × 60 mm^2^ with two thicknesses of 0.5 and 1.5 mm. The choice of the 96% alumina substrate is due to the use of a ceramic microelectronic material, low cost, and the sufficient vacuum density of the substrate obtained via tape casting production. Alumina ceramics comprising 96% of the material yield good predictable adhesion to pastes used, which also contain glass. There is a slight advantage in the speed of laser micromilling (the latter material is harder), which becomes more sensitive when producing a substrate with 80 packages, as presented in Figure 4. We rejected the use of 94.4% alumina pink color ceramics due to darkening of the color when we fired one in the kiln in air at 950 °C, although it is possible to use this type of ceramics in described technology.

The software and hardware developed for the micromilling process are described in more detail [11]. At the stage of 3D modeling, it is necessary to add jumpers to the model, which will hold it in the substrate (frame) array. The point of contact of the jumper with the 3D model depends on the size of the model; for our models, presented in Figure 2b, it is a pyramid with a vertex in the form of a square of 0.15 × 0.15 mm (where it is attached to the chip). The preparation time for milling a 3D object of this size with specialized software is a few minutes. The process of laser micromilling of Al_2_O_3_ ceramics was carried out at a speed of ~40 mm^3^/h. Depending on the required final ceramic surface quality, the milling speed can be changed up or down. After starting micromilling, the process can be paused at any time to view the milled object using a microscope with 400–2000× magnification or to measure the roughness/height of the milled layer using a point laser profiler integrated into the adaptive laser micromilling unit, and then continue milling from the stop point. Time of fabrication of both part of the package is 86 min (14 min takes a milling bottom part of the package from a 0.5 mm thick ceramic substrate, and 72 min takes a cap part of the package from a 1.5 mm thick ceramic substrate). Figure 4 demonstrates the result of ceramic laser micromilling—the substrate frame with finished caps and another substrate that only has contact pads and via holes for future deposition of silver thick film. This can be used for metallization by firing in the kiln to create the future bottom part of the package. After the first stage of laser cutting, silver paste was deposited and fired to form the contact pads and seal the vias. After firing of metallization, the second stage of laser micromilling followed, where the bottom 3D geometry of the package was formed.

### 2.3. Final Assembling of Sensor

**Figure 5:**
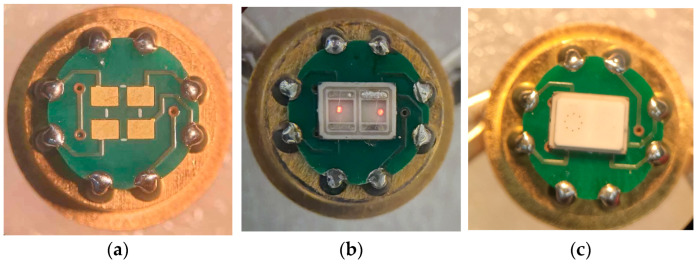
Final assembling of fabricated sensor before thermal conductivity tests: (**a**) The TO-8 holder [17] with soldered PCB interconnector for SMD sensor package; (**b**) Soldered to PCB bottom part of package with welded Pt-wire microheaters. The beginning of the heaters’ luminosity (approximately 600 °C) corresponds to 117 mW power consumption; (**c**) The photo with assembled sensor in order to test in vacuum laboratory facility for thermal conductivity measurement.

The final assembly of the sensor was carried out as follows. First, a spiral of platinum coated with glass was welded to a package base separated from the frame with fired metallization, and then electrical tests were carried out for the integrity of the contacts. Then, the cap package with deposited glass layer as glue was connected to the package bottom part and fired in the air atmosphere in muffle furnace. It was possible to manually add a little silver paste with a needle and fire the metallization back to restore full electrical contact with the holes. Another feature was that the microheater was welded with a spiral upward. This was necessary due to the specifics of the rapid production of the bottom part of package—the thinner the ceramic substrate, the faster it is milled by laser (often, this is directly proportional to the dependence of the production time on the volume of material removed). It is more profitable to make a thin base of the package and then choose a thicker cap for it. The thinness of the bottom part is also related to the diameter of the vias. The thinner the initial substrate, the smaller the initial diameter of the vias, and the easier it is to fill them with paste to create a hermetically sealed metallization of the vias. Also, the package cap does not contain metallization, so it is easier to manufacture—there is no need to perform additional technological operations with deposition and firing of conductive paste; it is enough to find the optimal dimensions of the internal cavity of the cap so that the platinum microheater is at the required distance from the walls of the package. In the final assembly to perform future thermal conductivity tests, assembled package of the manufactured sensor was soldered onto a PCB board installed on base of convenient standard glass-steal TO-8 package [17]. The photo with sensor ready for testing is presented in Figure 5c.

## 3. Results

The results can be divided into two sections: technology and operating characteristics of fabricated sensors. The interpretation of technology aspects is based on the concept of fast prototyping because time is a main non-renewable resource, and the price of a mistake is, first of all, lost time. Regarding technology aspects, there is equipment availability having an argent role. The interpretation of measurement properties of the fabricated sensor is based on the hypothesis that if the fabrication tech process temperature is 950 °C, and the resistance and tightness characteristics of the sensor do not change, then the sensor remains functionally operational. The properties of the sensor are tested at room temperature, but in a vacuum laboratory bench where the manufactured sensor experienced multiple changes of both low and high pressure, which confirms its functional properties when operating under harsh environmental conditions.

### 3.1. Technology Fabrication Aspects

When the sensor was designed, it was necessary to ensure the following aspects in parallel and sequentially:

- Platinum wire welding on metallization;
- Creation of sealed vias by metallization;
- Elimination of manual labor in the manufacture of a batch of sensors;
- Selection of metallization, which is soldered with standard tin, that contain solders.

**Figure 6:**
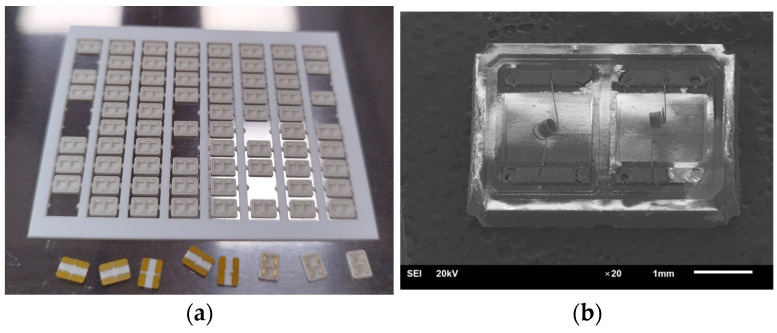
The manufactured bottom of the package with Au metallization: (**a**) The photo of substrate frame with bottom part of package before deposition of Au paste—separate packages with wet Au past thick film and already fired packages with gold metallization; (**b**) The image of bottom part welded to Au metallization Pt-wire microheater obtained via JEOL JSM-6610LV Scanning Electron Microscope. In place of via holes, it is possible to observe cracks of metallization.

This problem was solved in two iterations, and in the following way, the design of the case was initially chosen, on which the gold conductor paste of the PZL-M [18] brand was manually deposited. This is a relatively expensive 82% gold content product for experiments that provided a guarantee of solderability with lead–tin solders. It is widely used for the manufacturing of conductors with the stencil method printing on ceramics VK-94-1 (pink ceramics with 94.4% Al_2_O_3_ [19]) in the manufacture of ceramic-based hybrid microassemblies. When manufacturing the bottom of the package, the stage without the screen printing paste was skipped, and the finished bottom of the package was immediately produced using a laser (to micromill two combined STL files in parallel with contact pads and 3D geometry of the package) in the dimensions corresponding to the model in Figure 2b. Indeed, there were several variations of the package that differed in the level of contact pads (the pads were either convex or remote). It was experimentally established that for manual labor, the lower contact pads should be depressed, and the upper ones, on the contrary, should protrude above the body for the convenience of contact welding of a platinum microwire. Several options for adapting the model of the original sensor were made within 45 min, and manual application of the gold paste on both sides of the body was tested (with quick drying of each layer before subsequent deposition). The gold metalization was fired at 850 °C for an hour, close to the temperature profile reported in [18]. An electric vacuum furnace was used for pressing and firing ceramics [20], which has a 3-inch table that accords in dimension with our 60 × 48 mm Al_2_O_3_ substrate. After firing, contact welding of the platinum microwire was carried out, which showed excellent results (welded in first attempts). After welding, an electrical test was carried out via light ignition of a microheater spiral similar to that shown in Figure 5b. Also, tests for soldering the package contact pads with lead–tin solders showed excellent results, but for gold, this was expected. The initial concept showed its efficiency but revealed problems with the cracking of vias covered by gold metallization, which are present in the SEM image in Figure 6b. This problem was solved in the second iteration. Moreover, the solution had to be radical since hand-made deposition did not guarantee high-quality sealing. When using gold paste, the appearance of sealing could be achieved due to the formation of a meniscus; when burned, the paste is pulled out due to surface tension and covers the hole—we needed a thick conductive monolithic film that could withstand the pressure drop and completely fill the vias hole.

**Figure 7:**
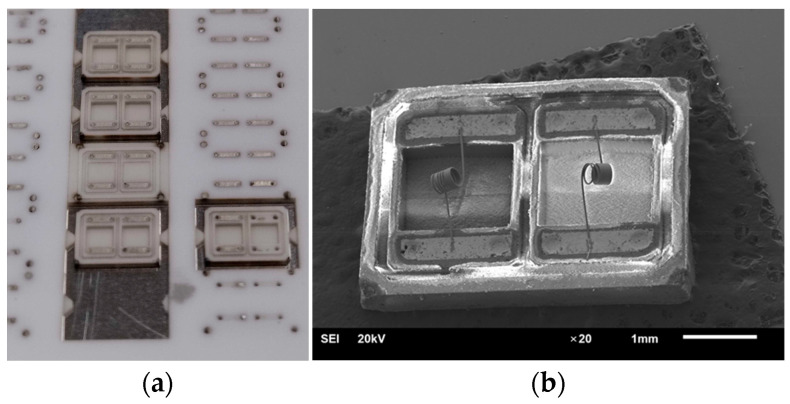
The manufactured bottom of the package with Ag metallization: (**a**) The photo of substrate-frame after laser micromilling of 3D geometry bottom part of package with Ag metalization; (**b**) The image of bottom part welded to Ag metallization Pt-wire microheater obtained using JEOL JSM-6610LV Scanning Electron Microscope. In place of via holes, it is not possible to observe cracks of metallization, and only total filling of vias holes with Ag metallization.

Our next iteration was carried out using silver conductive paste PP33 [21] brand, the cost of which is an order of magnitude lower than gold. Therefore, we could afford more metallization material consumption. The selected high-platinum-content silver paste is characterized by high dealloying resistance, low silver migration, and high solderability. The manufacturing process was carried out according to Figure 1a. The paste for forming metallization was applied to pre-engraved cavities of contact pads with a depth of 30 μm, between which vias were punched with a laser. Filling with paste was carried out with the squeegee of a screen printing machine without using a mesh stencil. This approach saved a significant amount of time, allowing you to change the topology literally immediately during laser production of the workpiece and without thinking about additional equipment (making a photomask, tensioning the stencil, etc.). The paste was screen-printed twice on each side of the substrate with a drying interruption. This approach was necessary for greater compaction of the paste in the vias. The traces of paste remaining on the substrate after drying were removed with an alcohol wipe. Cleaning with an alcohol wipe before firing the paste may not be necessary, but then the time for subsequent grinding increases many times over. It is also necessary to take into account surface unevenness and ceramic substrate roughness. It may be ideal to deposit the paste on an already polished ceramic substrate, where all unevenness is leveled out in advance, the adhesion of the paste is weak, and it can be easily separated from the substrate, even after burning. We reached this understanding after performing all the experiments; we also need to take into account that the cost of a polished substrate is an order of magnitude higher than a standard one (surface so-called “as fired”). The final removal of not using metallization spots is necessary to guarantee the purity of subsequent laser micromilling—the presence of a conductive phase that is weakly oxidized in the air due to the deposition of laser ablation vapors can create traces for weak current leaks given the miniature size of the sensor package design. After firing in accordance with the temperature profile specified in [21], traces of the remaining metallization on the surface of the substrate were removed using a Mecatech 234 grinding and polishing machine. Also, processing with a grinding and polishing machine made it possible to level the level of the contact pads with the ends of the package, since when firing in the metallization, standard for thick films shrinkage occurred. The result of high-quality sealing of vias can be observed in the SEM image of Figure 7b.

Silver metallization requires the development of the welding mode; apparently, the instability of the welding is associated with the issue of mechanical cleaning of the contact pads following laser micromilling. Sometimes, too much power when welding a microwire can evaporate the contact pads, and not enough power can weld the wire to the welding electrode, so more test samples were required than with gold metallization. After welding the microheater, the bottom part of the package was connected to the cap of the package by using PD-11 glass paste with a maximum firing temperature of 850 °C [22]. During the sealing process, we put some weight onto the “sandwich” with the ceramic part of the bottom and cap of the sensor for better adhesion.

### 3.2. Thermal Conductivity Test

To perform thermal conductivity tests, the assembled package of the manufactured sensor with silver metallization was soldered onto a PCB board (photo with results can be seen in Figure 5). The PCB board, in turn, was soldered to the TO-8 package [17], which had the required number of pins for electrical connections. It is difficult to connect the package electrically to a direct SMD package due to its small dimensions. For high-temperature measurements, the organic PCB must be replaced with a ceramic one, and the soldering must be replaced by firing with conductor paste.

For testing of the sensor, a vacuum laboratory facility for thermal conductivity measurement was assembled, which is presented in Figure 7, consisting of a cylinder with 100% nitrogen (B1), a cylinder with 100% helium (B2), two gas reducers (RG1, RG2), four manual valves (VP1–VP4), a BP-3 gas mixer (A), a vacuum chamber (CV), a DS18B20 digital temperature sensor (TE) (SAMIORE Robot Ltd., Hong Kong, China), an MS5611 digital pressure sensor (PT1) (Measurement Specialties Ltd., Shenzhen, China), a pointer pressure vacuum gauge (PT2), and an oil-diffusion vacuum pump (ND). The gas lines are made of a flexible gas hose; the PT2 pressure gauge is connected via a tee.

**Figure 8:**
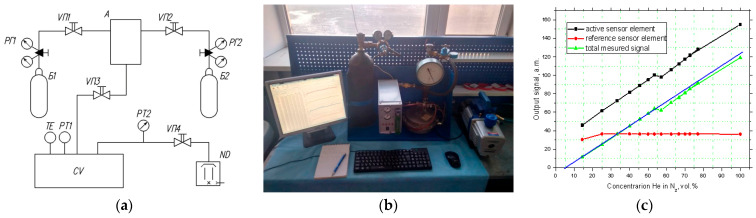
Thermal conductivity test for fabricated sensor: (**a**) Schematic diagram of a vacuum laboratory facility used for thermal conductivity measurement; (**b**) Photo of laboratory facility during measurements; (**c**) Plot with result of measurement for helium and nitrogen binary mixtures as a function of power consumption by microheater at constant working temperature of 400 °C for both sensor elements (the temperature of gas mixtures is 20 °C). The blue line is linear approximation of total measured signal for thermal conductivity sensor as a difference output signal from active and reference sensor elements.

The experiments were carried out using a nitrogen–helium mixture obtained using a BP-3 gas mixer from two cylinders with 100% test gases (helium and nitrogen). The BP-3 device allows you to create 16 combinations of mixtures with different proportions of gases. Tests were carried out for all 16 mixture combinations. During the experiments, the following were recorded: the pressure in the chamber using the MS5611 sensor, the temperature in the chamber using the DS18B20 sensor, and electrical signals from the thermal conductivity sensor. The measured signals were transmitted by UART protocol via a COM port to a PC, where, using the macros for the EXEL program, they were automatically entered into a table for further work with the collected data. A plot with measured results is presented in Figure 8c. The output signal from the sensor is linear in the range of 15–100% vol. of helium. The comparative element is sealed and operates stably over the entire range.

## 4. Discussion

The results demonstrate the effectiveness of the developed technology. Problem questions include the limiting temperatures’ values for the fabricated prototype of the thermal conductivity sensor. We proceeded from the hypothesis that if the fabrication tech process temperature is 950 °C, and the resistance and tightness characteristics of the sensor do not change, then the sensor remains functionally operational. Creating a stand for testing sensor operation at high temperatures is beyond the scope of this technological work and is a separate, complex, and time-consuming task. To solve this problem, you can use the methods described in [23]. The findings and their implications will be discussed in the broadest context possible in our next work dedicated purely to thermal conductivity measurement during high-temperature tasks. Future research directions may also be highlighted, which will be interesting in the context of micromachining.

Perspective future research directions may be based on increasing the working temperature of the sensor. This may be achieved by using more advanced materials stable at high temperatures. For example, researchers may start developing a strategy described by the company Kyocera in [24] as a “Hermetic Ceramic Package for High Temperature Application”. The feature of the Kyocera package is platinum metallization, which enables wire bonding between the semiconductor die and package. A heat resistance test (1100 °C × 3 h) has been conducted and verified no degradation compared to the wire bonding strength before and after the heat-resistance test. Therefore, 1100 °C is not the limit, especially considering that the method of manufacturing microwires is casting at platinum melt temperatures of 1768.3 °C during the “Taylor-Ulitovski process” where wire glass isolation viscosity is still high. For thermal-conductivity measurements, it is also important to have a microheater temperature 200 °C higher than the ambient temperature (this is our experience for good resolution of measurement); that is, temperatures of 1300 °C in the working environment are achievable without any complex physical phenomena. Technological manufacturing temperatures for a high-temperature sensor can be in the range of 1500–1600 °C. If discussing package temperature stability, it is possible to orientate on melting point for Al_2_O_3_ 2044 °C or alternative ceramics ZrO_2_ with 2715 °C polycrystalline base pure material, but the real working and certainly technology process temperature will be lower because ceramic materials need to take into account the glass phase, which binds the polycrystalline base of the ceramic substrate. The weakest link of our tech process is the metallization of the package. If, in the current work, the metallization was made of silver (melting point 961.9 °C) and gold (melting point 1064.2 °C), materials compatible with LTCC technology, then in the future, it is necessary to use platinum, and depending on the temperature of the technological process, it is necessary to select the composition of the paste with a certain temperature property of the glass. It is probably better to use accessible commercial platinum paste for the HTCC process fabrication of a lambda probe based on ZrO_2_ ceramics [25] with a firing temperature of 1500 °C. From the perspective of the technological process, it is also necessary to update the fabrication equipment used—only a furnace for firing metallization. For example, researchers could use the MTI KSL-1800X-S compact muffle furnace [26] with an upper temperature of 1800 °C.

## 5. Conclusions

The main aim of this paper was to demonstrate a rapid prototyping method for thermal gas sensors in a miniature ceramic SMD package and the reproducibility and stability of manufactured sensor properties based on use during high-temperature technology processes. With these fabricated thermal gas sensors, we used the conjunction between state-of-the-art technologies and materials: perfect mechanical glass-coating Pt-wire fabricated using the Taylor–Ulitovski technology process, sealing via holes using thick film metallization printed without a mask, and qualitative geometrical parameters of bulk ceramic package parts fabricated using laser micromilling. For the present article’s conception, low-scale thermal gas sensor production should be cost-effective and should not require any clean-room technology. The described technologies for thermal conductivity gas sensor fabrication have the potential to be used in another sensor application (primarily in applications where there are high temperatures and contact of sensor elements with the environment) with the same possibilities of reducing the production costs of the final product and simplifying and speeding up the whole production process. The described approach to the development of sensors can lead to new opportunities for advanced applications in different gas-sensing tasks, such as aerospace and industrial applications—when there are high temperature fluctuations, pressure drops, and aggressive ambient environments. 

## Data Availability

The data presented in this study are available on request from the corresponding author.

## References

[1] Gardner: E.L.W., Gardner J.W., Udrea F. (2023). Micromachined Thermal Gas Sensors—A Review. Sensors.

[2] SGX Sensortech VQ5 Series Thermal Conductivity Gas Detector Elements. https://www.sgxsensortech.com/content/uploads/2014/07/VQ51-VQ5-Thermal-Conductivity-Pellistor-Pair-%E2%80%93-2v_175mA1.pdf.

[3] SGX Sensortech VQ500 Series EU Declaration of Conformity EUD-0066. https://www.sgxsensortech.com/content/uploads/2014/08/EUD-0066-VQ500-DoC-Special-Instructions-V2.pdf.

[4] RS Product Solutions. https://www.rsproductsolutions.nl/Thermal-Conductivity-Gauge/.

[5] Neroxis MTCS 2200—Gas Micro Thermal Conductivity Sensor for Gas. https://www.neroxis.ch/wp-content/uploads/2020/04/DPM-18-090-002-E-MTCS-2200-Micro-Thermal-Conductivity-Sensor-for-Gas_Brochure_EN.pdf.

[6] JSC Plant “Mars” Metal-Glass Packages of Type KT-1, KI-1(TO-18). https://z-mars.ru/produktsiya/korpusa-pp/metallosteklyannye/korpusa-metallosteklyannye-tipa-kt-1-ki-1to-18.html.

[7] Samotaev N., Dzhumaev P., Oblov K., Pisliakov A., Obraztsov I., Ducso C., Biro F. (2021). Silicon MEMS Thermocatalytic Gas Sensor in Miniature Surface Mounted Device Form. Chemosensors.

[8] Kim M.-S., Nishikawa H. Thermal stability of electroless nickel/immersion gold surface finish for direct bond copper. Proceedings of the 5th Electronics System-Integration Technology Conference (ESTC).

[9] Sešek A., Makarovič K. (2022). Metallization, Material Selection, and Bonding of Interconnections for Novel LTCC and HTCC Power Modules. Materials.

[10] Samotaev N., Litvinov A., Oblov K., Etrekova M., Podlepetsky B., Dzhumaev P. (2023). Combination of Material Processing and Characterization Methods for Miniaturization of Field-Effect Gas Sensor. Sensors.

[11] Samotaev N., Oblov K., Dzhumaev P., Fritsch M., Mosch S., Vinnichenko M., Trofimenko N., Baumgärtner C., Fuchs F.-M., Wissmeier L. (2021). Combination of Ceramic Laser Micromachining and Printed Technology as a Way for Rapid Prototyping Semiconductor Gas Sensors. Micromachines.

[12] Karelin A., Baranov A.M., Akbari S., Mironov S., Karpova E. (2019). Measurement Algorithm for Determining Unknown Flam-mable Gas Concentration Based on Temperature Sensitivity of Catalytic Sensor. IEEE Sens. J..

[13] Ivanov I.I., Baranov A.M., Talipov V.A., Mironov S.M., Akbari S., Kolesnik I.V., Orlova E.D., Napolskii K.S. (2021). Investigation of catalytic hydrogen sensors with platinum group catalysts. Sens. Actuators B Chem..

[14] Karpova E., Mironov S., Suchkov A., Karelin A., Karpov E.E., Karpov E.F. (2014). Increase of catalytic sensors stability. Sens. Actuators B Chem..

[15] Somov A., Baranov A., Suchkov A., Karelin A., Mironov S., Karpova E. (2015). Improving interoperability of catalytic sensors. Sens. Actuators B Chem..

[16] Zhukova V., Corte-Leon P., Blanco J.M., Ipatov M., Gonzalez-Legarreta L., Gonzalez A., Zhukov A. (2022). Development of Magnetically Soft Amorphous Microwires for Technological Applications. Chemosensors.

[17] JSC Plant “Mars” Metal-Glass Packages of Type 3301.8 (TO-18). https://z-mars.ru/produktsiya/korpusa-datchikov/korpusa-metallosteklyannye-33018-to-8.html.

[18] Elma-Pastes LLC Conductive Au Pastes. https://elma-paste.ru/paste_au/.

[19] GN Electronics Ceramic Substrates Based on Aluminum Oxide (Al_2_O_3_). https://www.gnelectronics.ru/produktsiya/keramicheskie-podlozhki/keramicheskie-podlozhki-na-osnove-oksida-alyuminiya-al2o36027123/.

[20] AVERON Scientific and Production Complex Electric Vacuum Furnace for Pressing and Firing Ceramics. https://www.averon.ru/catalog/elektrovakuum__pechi/evp_press_2_0/.

[21] Elma-Pastes LLC Conductive Pt Pastes. https://elma-paste.ru/paste_pt/.

[22] Dielectric Insulating Pastes. http://www.depa.ru/pd.htm.

[23] Rothman A.J., Bromley L.A. (1955). High temperature thermal conductivity of gases. Ind. Eng. Chem..

[24] Kyocera Corporation Surface Mount Ceramic Packages for Electronic Devices. https://global.kyocera.com/prdct/semicon/semi/smd_pkg/.

[25] Vibrantz Technologies Electronic Materials. https://www.ferro.com/products/product-category/electronic-materials/htcc-system-materials/high-temperature-co-fired-ceramic-materials/htcc-nox-sensor-systems-and--materials.

[26] MTI Corporation 1800C Compact Muffle Furnace. https://www.mtixtl.com/mufflefurnace-KSL-1800X-KA-S-UL.aspx.

